# The influence of achievement goals on objective driving behavior

**DOI:** 10.1371/journal.pone.0276587

**Published:** 2022-10-27

**Authors:** Martin Nicolleau, Nicolas Mascret, Claire Naude, Isabelle Ragot-Court, Thierry Serre

**Affiliations:** 1 Aix Marseille Univ, CNRS, ISM, Marseille, France; 2 TS2-LMA, Univ Gustave Eiffel, IFSTTAR, Salon de Provence, France; IZA - Institute of Labor Economics, GERMANY

## Abstract

Investigating psychological characteristics through self-reported measures (e.g., anger, sensation seeking) and dynamic behaviors through objective measures (e.g., speed, 2D acceleration, GPS position etc.) may allow us to better understand the behavior of at-risk drivers. To assess drivers’ motivation, the theoretical framework of achievement goals has been studied recently. These achievement goals can influence the decision-making and behaviors of individuals engaged in driving. The four achievement goals in driving are: seeking to improve or to drive as well as possible (mastery-approach), to outperform other drivers (performance-approach), to avoid driving badly (mastery-avoidance), and to avoid being the worst driver (performance-avoidance). Naturalistic Driving Studies (NDS) provide access to the objective measurements of data not accessible through self-reported measurements (i.e., speed, accelerations, GPS position). Three dynamic criteria have been developed to characterize the behavior of motorists objectively: driving events, time spent above acceleration thresholds (longitudinal and transversal), and the extent of dynamic demands. All these criteria have been measured in different road contexts (e.g., plain). The aim of this study was to examine the predictive role of the four achievement goals on these objective driving behaviors. 266 drivers (96 women, 117 men) took part in the study, and 4 242 482 km was recorded during 8 months. Simultaneously, they completed the Achievement Goals in Driving Questionnaire. The main results highlighted that mastery-approach goals negatively predicted hard braking and the extent of dynamic demands on plain and hilly roads. Mastery-approach goals seem to be the most protective goals in driving. Future research on the promotion of mastery-approach goals in driving may be able to modify the behavior of at-risk drivers.

## Introduction

In Europe, 80 559 people died on the road in 2016 [1], and it is estimated that 3 million people are injured on the road each year. In France, 3,239 people were killed on the road in 2019, including 1,621 motorists [2]. Road accidents are the leading cause of death for 15-24-year-old. The loss of life and serious injuries are socially disastrous, and they represent important economic costs to society (i.e., financial compensation). Consequently, road safety represents a major societal issue. Blanco [3] estimated that human error is involved in 70–90% of road accidents. Numerous studies [4] have assessed self-reported psychological characteristics, such as sensation seeking [5] and anger [6], to better understand self-reported driver behavior. New measures have also recently been included in the literature through Naturalistic Driving Studies (NDS), which focus on real-world driver performance and behaviors [7]. These studies have used Event Data Recorders (EDR) to collect data on vehicle dynamics [8, 9]. This kind of measure assesses objective driver behavior. Self-reported psychological variables have already been connected to these objective measures, but achievement goals have not hitherto been examined, although their interest in the driving domain has recently been highlighted [10, 11].

### The achievement goal theory

The achievement goal theory has been widely used in the literature to assess the motivations of individuals engaged in an achievement context. The work of Maehr and Nicholls [12] and Dweck and Elliot [13] form the basis of this theory. Achievement goals can be defined as how an individual values demonstrating competence or avoiding demonstrating incompetence in relation to himself/herself or others. There is an important distinction to be made between the goal setting theory [14], in which the setting of objectives such as to achieve exam is studied, and achievement goals theory in which the motive of this objective, why the person seeks to achieve his/her exam, is investigated. This theoretical framework is based on the fact that individuals are influenced by intention and guided by a goal that they will rationally pursue [10]. Therefore, achievement goals can influence the decision-making and behaviors of individuals engaged in an achievement context. This context can be defined as a situation in which self-competence is assessed, the result depends on the individual, success is uncertain, and success is socially valued [15]. Driving is an achievement context [10], as the self-competence is continuously evaluated by the driver, passengers and other road users. The driver is the principal responsible for his/her behavior. Success is uncertain, and it depends on numerous parameters (e.g., the driver behavior, other road users, the car, road conditions, weather conditions), and even a good driver can have an accident. Finally, being a good driver is always socially valued, especially by friends and family, while being a bad driver can be socially devalued.

In the 1980s, the first researchers at the origin of this theoretical framework [12, 13, 16] developed the « Dichotomous model ». These researchers distinguished between mastery goals and performance goals. Mastery goals refer to an individual who wants to show his/her competence by mastering the task, while performance goals refer to an individual who wants to show his/her competence in relation to others. In the 1990s, Elliot & Harackiewicz [17] developed the « Trichotomous model ». The authors applied the notion of approach motivation (i.e., behavior oriented towards positive consequences) and avoidance motivation (i.e., behavior oriented towards the avoidance of negative consequences) to performance goals. The model is composed of three types of goals, the mastery goals which refer to an individual who wants to develop his/her competence and control the task, the performance-approach goals which refer to an individual who wants to show superiority over others, and the performance-avoidance goals which refer to an individual who wants to avoid showing incompetence over others. A few years later, Elliot [18] and Pintrich [19] proposed the 2x2 model of achievement goals. This model, consisting of four goals (i.e., mastery-approach, mastery-avoidance, performance-approach, and performance-avoidance), was the most used in different domains such as education [20] and sport [21]. The study of achievement goals in driving is relatively recent. Mascret et al. [10] were the first to develop a scale for assessing achievement goals in driving. In driving, the four achievement goals represent: a driver who wants to drive better and better and master the driving task (mastery-approach goal), a driver who wants to outperform others drivers (performance-approach goal), a driver who avoids driving badly (mastery-avoidance goal), and a driver who avoids driving worse than others (performance-avoidance goal). In the literature of various domains (e.g., education, sport, work, driving), research has shown that mastery-approach goals are primarily related to positive outcomes in terms of performance, intrinsic motivation and learning strategies [22, 23]. In driving, mastery-approach goals positively predict interest in driving [10]. Results are more contrasted for mastery-avoidance goals. In education, mastery-avoidance goals were found to be negatively related to interest and performance [23, 24], whereas in sport mastery-avoidance goals were not negatively related to performance [23]. In driving, mastery-avoidance goals were found to be negatively related to self-reported accidents, self-reported at-fault accidents, and violations [10, 11]. Surprisingly, mastery-avoidance goals seem to have a protective role in the driving context. Similarly to mastery-avoidance goals, the results of studies conducted on performance-approach goals are contrasted. In Payne et al.’s [25] meta-analysis, performance-approach goals are related to both positive outcomes (i.e., performance) and negative outcomes (i.e., anxiety). Contrary to other domains, performance-approach goals in driving are related to negative outcomes only (i.e., sensation seeking, aggressive violations, and ordinary violations). This is less contrasted for performance-avoidance goals in different contexts [23]. Performance-avoidance goals are related to non-adaptative outcomes (i.e., anxiety, negative affect, disinterest, low performance). Consistent with these results, performance-avoidance goals in driving are positively related to aggressive violations [11].

The driving behavior variables related to achievement goals in Mascret et al. [10, 11] studies were self-reported measures, which improves knowledge of driving behavior through low-cost and easy-to-collect data from large samples. However, self-reported measures can be subject to recall bias [26], social desirability [27] and participant rough estimates [28]. Objective measures obtained by the NDS provide valid and more accurate measures of driving behavior [29], because they are obtained in normal driving conditions with validated sensors. Furthermore, these studies give access to data unattainable through self-reported measures, such as speed, acceleration, braking, GPS position, driving context (road type and relief), crashes, or events [30]. To date, achievement goals have not been investigated in NDS.

### Naturalistic driving studies

Initially in the 1970s [31], driving simulators were widely used to assess objective driver behavior, but it is sometimes difficult to transpose the results obtained with simulators into normal driving conditions [32]. In a second step in the 2000s [33], Event Data Recorder (EDR) and smartphone applications were developed to continuously record driver behavior (speed, acceleration, deceleration, GPS) under real driving conditions. This has led to the development of Naturalistic Driving Studies (NDS). These studies consist in equipping volunteer cars with an EDR or a smartphone application, which will record the dynamics continuously, from a few months to several years [33]. These studies provide access to natural driver behavior, to study crashes, near-crashes, and incidents. The first NDS was the U.S 100-car driving study, conducted by Dingus et al. [33]. Afterwards, many NDS have been conducted worldwide, for instance the Australian 400-car naturalistic driving study [34], UDRIVE in Europe [35], the Shanghai Naturalistic Driving Study in China [36], the Japan Naturalistic Driving Study by the Japan Automobile Manufacturers Association [37], the Saving Lives through Road Incident Analysis Feedback project (SVRAI) in France [38]. In 2020, Singh and Kathuria [39] identified a total of 135 NDS in the literature.

From the objective data collected in the NDS, variables have been developed to characterize driver behavior, such as driving incidents, extent of dynamic demands, and percentages of time spent above acceleration thresholds.

In general, it is difficult to assess driver behavior during crashes because of the rarity of these events. Therefore, near-crashes and incidents are often studied [33]. Wu et al. [40] observed a positive relationship between crashes, near-crashes, and crash-relevant incidents (i.e., “an event in which a crash was avoided by extreme steering or braking input (or both) but did not approach driver or vehicle limits” p.214). Thus, incidents are a relevant variable to better understand driver behavior. Naude et al. [9] measured 338 incidents on 51 vehicles during a one-year recording period, and detected no crashes. They estimated a ratio of 1 crash per 20,000 incidents. To detect incidents, they used longitudinal and lateral acceleration, speed, and jerk (i.e., derivative of acceleration). According to Bagdadi [8], the jerk can be used to detect driving events. A ratio of the number of kilometers between each incident can be calculated. The higher the ratio is, the less likely the driver is exposed to risky driving situations.

To study the dynamic demands generated by drivers with their vehicle (i.e., acceleration/braking, right/left turn), Lechner and Naude [41] developed a criterion called “Travel synthesis”. At every moment of every trip, the crossing of longitudinal and transversal acceleration values is counted and recorded in a matrix in 1 m/s^2^ intervals. The number of different crossings reached (the area of the 2D graph) gives an indication of the dynamic load levels and, in particular, the extreme values reached at least once by the driver. The more a driver explores the high dynamic capabilities of his vehicle load, the larger the area of the acceleration crossing will be. Liu et al. [42] used a bivariate distribution of accelerations. This distribution also illustrates the extent of dynamic load, but measuring its surface is more difficult.

To assess high acceleration, braking and cornering demands, Lechner and Perrin [43], and Naude et al. [9] used percentages of time spent above longitudinal or transversal acceleration thresholds. The authors reported that it is very rare for drivers to exceed 0.3g in transversal acceleration (only 2% of the time) and even rarer in longitudinal acceleration (0.4%-0.5%). Individuals who exceed these time percentages would be demonstrating "sporty" driving, which may be risky driving depending on the driving situation.

To investigate driving behavior, it may be relevant to consider the driving environment, and more particularly the type of road (i.e., urban, extra-urban, highway), but also the relief on which the road is situated (i.e., plain, hill, mountain). Indeed, Lechner and Perrin [43] observed that on mountain roads, individuals commit stronger transverse accelerations than on plain roads. Other studies such as Xu et al. [44], Si et al. [45] were also interested in the influence of relief on driving behavior, and observed that behavior varies significantly between plains and mountains. The consideration of relief would significantly improve the knowledge on driving behavior.

Recently, real-world driver behavior has been studied in relation to personality traits or characteristics, such as anger [46], openness to experience, conscientiousness, extraversion, agreeableness, and neuroticism [47, 48]. Achievement goals have not been investigated to date in relation to these new objective measures.

### Achievement goals and objective measures

Van Yperen et al. [23] in their meta-analysis have investigated the relationships between objective performance and achievement goals in three domains (work, sport, and education). They showed that, in general, performance attainment was positively associated with performance-approach and mastery-approach goals, whereas it was negatively associated with performance-avoidance and mastery-avoidance goals. Although both performance-approach and mastery-approach goals are positively related to performance, only mastery-approach goals should be promoted because of the negative consequences of performance-approach goals in the social and ethical domains, such as cheating and dissatisfaction [23]. More specifically, Van Yperen et al. [23] showed the domain studied was a moderator of the relationships between achievement goals and performance. For example, a negative correlation was found between performance and mastery-avoidance goals in the education domain, whereas this relationship was not found in the sport domain. Therefore, it was relevant to examine these relationships in the driving domain with objective measures. This will improve knowledge about achievement goals and may or may not confirm the observed relationships with self-reported driving variables.

In the meta-analysis of Van Yperen et al. [23], performance in education, sport, and work was defined in a broad sense, including for example test scores, competition results, and evaluation by coaches, teachers, or supervisors. In the driving domain, defining a performance per se is complex. Driving performance can be considered as the behavior that results in the safest driving. It can be assessed on the basis of objective behaviors such as driving incidents, percentage of time spent above an acceleration threshold, or the extent of dynamic demands. But to date, no studies have been conducted to examine the relationships between achievement goals in driving and objective driver behaviors in the real world (i.e., incidents, percentage of time spent over an acceleration threshold, and extent of dynamic demands), which was the purpose of the study.

### Hypotheses

In education, sport, and work, mastery-approach goals are the most adaptative goals [23] and they predicted some positive outcomes in driving such as interest [10]. Therefore, we might expect mastery-approach goals to negatively predict incidents, extent of dynamic demands, and percentage of time spent over an acceleration threshold. Although performance-approach goals are sometimes related to positive consequences such as performance attainment [23], we might expect them to be positive predictors of incidents, extent of dynamic demands, and percentage of time spent over an acceleration threshold, given that in driving it has been observed to be negatively related to self-reported driving consequences [11]. Van Yperen et al. [23] observed that mastery-avoidance goals are contrastingly related to performance in education and sport, whereas in driving, it was observed using self-reported measures that these goals may have a protective role on accidents, at-fault accidents, violations and sensation seeking [11]. Thus, we expect mastery-avoidance goals to negatively predict incidents, extent of dynamic demands, and percentage of time spent over an acceleration threshold. Given that performance-avoidance goals are negatively related to performance achievement [23] and also related to negative driving consequences such as aggressive violations [11], we expect these goals to be positive predictors of incidents, extent of dynamic demands, and percentage of time spent over an acceleration threshold. The influence of road type and topographic context on these relations will be investigated. If a relationship between a type of achievement goals and significant transversal acceleration is observed, this will determine whether this relation is present in driving in all contexts, or whether it is due to a context where transversal accelerations are more frequent (e.g., mountain).

## Method

### Participants

266 French drivers (96 women, 170 men, *M_age_* = 42.08 years, *SD* = 11.38, range = 22–77 years) voluntarily and anonymously participated in the study. Only drivers with a category B license (i.e., French driving license for car) were included (*M_years of driving license_* = 21.83, years, *SD* = 11.81), with an average of 649 trips per drivers (*SD* = 377), an average driving time of 202 hours per drivers (*SD* = 100), and with a total mileage of 4 242 482 kilometers (*M_mileage of drivers_* = 15 949 km, *SD* = 9 099).

### Procedure

Participants to this study were selected on a voluntary basis, from a panel of people that Michelin regularly solicits to conduct studies on driving behavior. To measure driver behavior, the vehicles of the 266 participants were equipped with a specific data acquisition system (Dwilen) developed by DDI (Driving Data to Intelligence), a subsidiary of Michelin that exploits mobility data. This data collection system includes a GPS with a frequency of 1 Hertz, and a three-dimension accelerometer (i.e., x: transversal acceleration, y: longitudinal acceleration, and z: vertical acceleration) with an acquisition frequency of 5 Hertz. Driving data were collected continuously during 8 months, representing a total of approximately 53 730 hours of driving. Individuals commit to being the only ones to drive their equipped vehicle during the recording period. Participants also filled out the questionnaire assessing achievement goals in driving, after the 8-month recording period. They gave informed consent and were not made aware of the purpose of the study. The study met the requirements of the institutional board of Aix-Marseille University and of the Commission Nationale de l’Informatique et des Libertés (n˚2004–801). The consent was requested and obtained in written form. The data were collected and analyzed completely anonymously.

### Measures

#### Achievement goals in driving

The four achievement goals in driving were assessed using the Achievement Goals in Driving Questionnaire (AGQ-D, [10]). Mastery-approach goals (e.g., “*When driving*, *my goal is to drive better and better*”), performance-approach goals (e.g., “*When driving*, *my goal is to perform better than others*”), mastery-avoidance goals (e.g., “*When driving*, *my goal is to avoid making mistakes*”), and performance-avoidance goals (e.g., “*When driving*, *my goal is to avoid driving worse than others*”) were assessed using a Likert scale ranging from 1 (completely disagree) to 5 (completely agree). Results of the confirmatory factor analysis were acceptable [49, 50]: *χ2* (48, *N* = 299) = 124.43, *p* < .001, CFI = .936, TLI = .912, SRMR = .053, RMSEA = .073. Using McDonald’s omegas, a good level of internal consistency [51] was found for mastery-avoidance (*ω* = .712) and performance-approach goals (*ω* = .837). The internal consistency of mastery-approach (*ω* = .693) and performance-avoidance goals (*ω* = .697) was somewhat low, but the value is very close to .700, which can be considered acceptable.

#### Longitudinal accelerations

Longitudinal accelerations were recorded continuously for each vehicle during the data collection period with a frequency of 5 Hertz. A distribution of longitudinal accelerations every 1 m/s^2^ was then obtained. Finally, the percentage of time during which each driver exceeded a longitudinal acceleration of 3 m/s^2^ was calculated. A high percentage indicates that the driver often performed high accelerations. The percentage of time each driver spent below -3 m/s^2^ longitudinal acceleration, indicating hard braking, was also calculated. A high percentage indicates that the driver was often performing hard braking. This threshold was used in the 100-Car study by Klauer et al. [52] and also by Lechner and Perrin [43] to assess heavy acceleration and braking.

#### Lateral accelerations

Lateral accelerations, representing the centrifugal force pushing the vehicle outward when cornering, were also recorded at a frequency of 5 Hertz and the percentage of time during which each driver exceeded a lateral acceleration of 3 m/s^2^ was calculated. This threshold has been used in various studies [43, 52] to assess high cornering loads. Right and left turns were dissociated. A high percentage indicates that the driver subjected his car to high stresses during cornering.

#### Incidents

Incidents are critical driving situations considered risky because the vehicle reaches high dynamic demands in the longitudinal lateral or combined directions. Incident detection is based on the work of Naude et al. [9]. We considered longitudinal acceleration, lateral acceleration, and jerk (derivative of acceleration).

The acquisition frequency in the present study is 5 Hz whereas Naude et al. [9] developed their criteria using a frequency of 100 Hz. At lower acquisition frequency, a signal with less information is recorded, and the thresholds used to detect incidents are no longer suitable. A specific study was conducted to compare data recorders at 100 Hz and 5 Hz. It allowed to adapt the thresholds so as to obtain the same incidents.

The thresholds used by Naude et al. [9] are:

- Speed < 80 km/h, and Acceleration norm (x and y) > 6 m/s^2^ and Jerk > 2 m/*s*^3^,
- Speed > 80 km/h and Acceleration norm (x and y) > 5 m/s^2^ and Jerk > 2 m/*s*^3^,
- Speed > 100 km/h and Acceleration norm (x and y) > 4 m/s^2^ and Jerk > 2 m/*s*^3^.

The thresholds used in the study are:

- Acceleration norm (x and y) > 6 m/s^2^ & Jerk norm (x and y) > 3 m/*s*^3^

The number of incidents for a driver depends on his or her mileage. To get a representative indicator of driving style, the total driving time (in minutes) was divided by the total number of incidents. A high ratio indicates that the driver has a relatively calm driving style. A low ratio indicates sporty or riskier driving style (i.e., distraction, novice driver …).

#### The extent of dynamic demands

**Fig 1:**
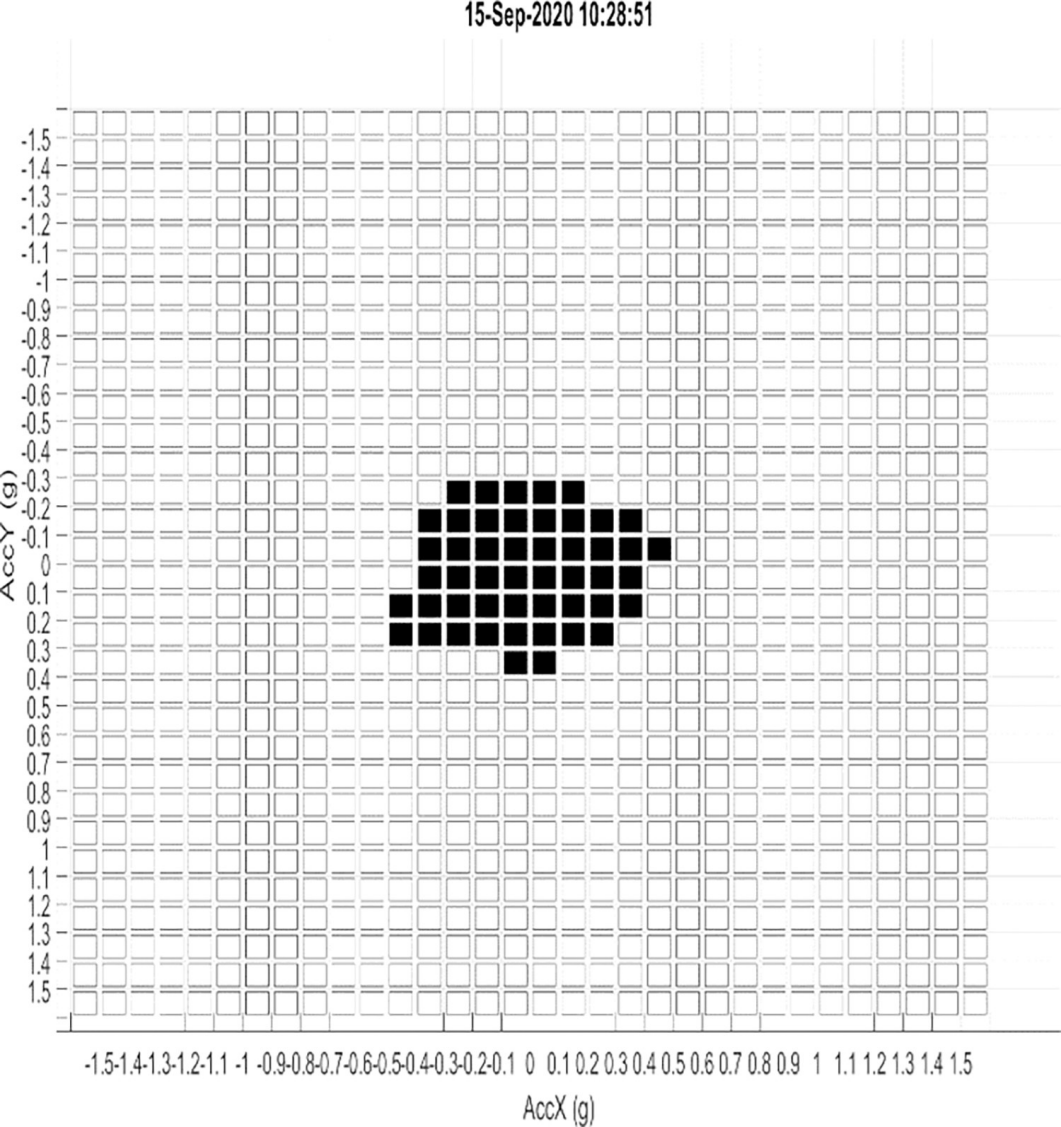
Exemple of the extent of dynamic demands.

This variable consists in crossing the lateral and longitudinal accelerations in a 2d matrix. The indicator used to characterize this extent is the percentage of filling of the matrix. A high value represents a driver who has strongly stressed his vehicle in acceleration, braking, and/or combined demands. For an illustration, see Fig 1.

#### Road contexts

Using the methodology developed by Michelin, we were able to know the type of road (i.e., urban, extra-urban, and highway) and the topography (i.e., plain, hill, mountain) at each instant of driving. All the variables which objectively characterize the driving behavior (i.e., incidents, percentage of time spent over an acceleration threshold, and extent of dynamic demands) have been measured in all these contexts.

### Data analyses

Mahalanobis distance at the multivariate level (*χ*^*2*^ (9) = 27.88, *p* < .001) was calculated to detect outliers [53]. To assess the univariate normality of the main variables, skewness (values ≤ |2|) and kurtosis (values ≤ |7|) were measured, following the recommendations of Curran et al. (1996) [54]. Then, descriptive statistics were conducted (see Table 1).

**Table 1:** Descriptive statistics of the final sample (without outliers), internal consistency, skewness, and kurtosis.

	Age	Years of driving license	Driving time (min)	Mastery-approach goals	Mastery-avoidance goals	Performance-approach goals	Performance-avoidance goals
Mean	42.08	22.83	7367.21	4.37	4.54	2.75	3.37
Standard deviation	11.38	11.81	4030.40	.542	.472	1.28	1.14
McDonald’s omega	-	-	-	.712	.708	.837	.703
Skewness	.843	.689	.370	-.675	-.682	.073	-.658
Kurtosis	.254	.181	-.009	.602	-.262	-1.27	-.429

Multiple regression analyses were performed to assess how the four achievement goals in driving predicted positive and negative longitudinal accelerations, lateral accelerations, incidents, and the extent of dynamic demands. Gender, age, years of driving license, and driving time were entered in the regression analyses to control for these variables.

## Results

### Preliminary results

No data were missing. 84 participants were detected as outliers and were excluded from the study, using the Mahalanobis distance [53]. The distribution of the main variables was approximately normal [54], based on measures of skewness (max = .843) and kurtosis (max = .602). 33 participants who did not drive sufficiently in the mountain context were removed from the analyses. Descriptive statistics, internal consistency, and skewness and kurtosis are presented in Table 1.

### Regression analysis

#### Main results

After controlling for gender, age, years of driving license, and driving time, the results of the regression analysis showed that mastery-approach goals are negative predictors of braking lower than -3 m/s^2^. They are also negative predictors of global extent of dynamics demands. In other terms, drivers who want to drive as well as possible (mastery-approach goals) generally brake less, and use the vehicle less dynamically. Performance-approach goals are positive predictors of longitudinal acceleration above 3 m/s^2^. This means that drivers who want to outperform other drivers generally accelerate more. Performance-avoidance goals are positive predictors of global extent of dynamics demands. In other terms, drivers who want to avoid being worse drivers than others generally use the vehicle more dynamically. The detailed results are presented in Table 2. Incidents and lateral acceleration were not predicted by any achievement goals (see Table 2).

**Table 2:** Results of the regression analyses.

	Accelerations > 3 m/s^2^		Braking < -3 m/s^2^		Extent of dynamic demands		Incidents		Right turn > 3 m/s^2^		Left turn > 3 m/s^2^	
	***R***	***R*** ^2^	***R***	***R*** ^2^	***R***	***R*** ^2^	***R***	***R*** ^2^	***R***	***R*** ^2^	***R***	***R*** ^2^
	.309	.095	.*257*	.*066*	.*698*	.*487*	.*356*	.*126*	.*298*	.*089*	.*224*	.*050*
	**Adj R^2^**	**Std error**	**Adj R^2^**	**Std error**	**Adj R^2^**	**Std error**	**Adj R^2^**	**Std error**	**Adj R^2^**	**Std error**	**Adj R^2^**	**Std error**
	.067	.196	.*037*	.*223*	.*471*	*1*.*06*	.*099*	*38*.*35*	.*061*	.*296*	.*021*	.*290*
**Model: Achievement goals**	***β***		***β***		***β***		***β***		***β***		***β***	
Gender	-.019		-.039		.030		-.029		-.132*		-.025	
Age	-.094		-.250		.090		.218		-.379*		-.004	
Years of driving license	-.029		.131		-.051		-.255		.201		-.173	
Driving time	-.187**		.094		.691***		.299***		-.078		-.191	
Mastery-approach goals	.136		-.150*		-.110*		-.130		-.118		.021	
Performance-approach goals	.221**		-.075		-.101		.028		-.034		.140	
Mastery-avoidance goals	-.037		.010		.100		-.039		-.009		-.044	
Performance-avoidance goals	-.126		.109		.146**		.059		.018		-.050	

Note.

*p < .05

**p < .01

***p < .001

#### Ancillary results

In this section, we present the analyses conducted with the road context. The results of the regression analysis showed that mastery-approach goals are negative predictors of the extent of dynamics demands in plain, of braking lower than -3 m/s^2^ in plain and also on hilly roads, and finally of right turn above 3 m/s^2^ on hilly roads (see Table 3). This means that drivers who want to drive better and better, brake less and use the vehicle less dynamically in plain, and brake less and take slower right turns on hilly roads.

**Table 3:** Results of the regression analysis for mastery-approach goals with the road context.

	Extent of dynamic demands in plain		Braking < -3 m/s^2^ plain		Braking < -3 m/s^2^ hilly		Right turn > 3 m/s^2^ hilly	
	***R***	***R*** ^2^	***R***	***R*** ^2^	***R***	***R*** ^2^	***R***	***R*** ^2^
	.693	.480	.*254*	.*064*	.*251*	.*063*	.*327*	.*107*
	**Adj R^2^**	**Std error**	**Adj R^2^**	**Std error**	**Adj R^2^**	**Std error**	**Adj R^2^**	**Std error**
	.464	1.051	.*035*	.*224*	.*034*	.*292*	.*079*	.*558*
**Model: MAP**	***β***		***β***		***β***		***β***	
Gender	.029		-.032		-.120		-.111	
Age	.091		-.249		-.094		-.349*	
Years of driving license	-.057		.129		.032		.207	
Driving time	.683***		.092		.053		-.082	
**Mastery-approach goals**	-.113*		-.153*		-.146*		-.224**	

Note.

*p < .05

**p < .01

***p < .001

Performance-approach goals are positive predictors of the extent of dynamic demands in plain. They are also negative predictors of accelerations above 3 m/s^2^ on plain and hilly roads, and of left turn above 3 m/s^2^ in plain (see Table 4). In other terms, a person who wants to outperform others drivers uses the vehicle less dynamically on plain, but accelerates more on hilly and plain roads, and takes the left turns more sharply on plains.

**Table 4:** Results of the regression analysis for performance-approach goals with the road context.

	Extent of dynamic demands in plain		Accelerations > 3 m/s^2^ plain		Accelerations > 3 m/s^2^ hilly		Left turn > 3 m/s^2^ plain	
	***R***	***R*** ^2^	***R***	***R*** ^2^	***R***	***R*** ^2^	***R***	***R*** ^2^
	.693	.480	.*308*	.*095*	.*261*	.*068*	.*242*	.*058*
	**Adj R^2^**	**Std error**	**Adj R^2^**	**Std error**	**Adj R^2^**	**Std error**	**Adj R^2^**	**Std error**
	.464	1.051	.*067*	.*200*	.*039*	.*252*	.*029*	.*289*
**Model: PAP**	***β***		***β***		***β***		***β***	
Gender	.029		.037		-.063		-.011	
Age	.091		-.091		-.124		.010	
Years of driving license	-.057		-.029		.009		-.194	
Driving time	.683***		-.189**		-.106		-.015	
**Performance-approach goals**	-.115*		.220**		.200**		.152*	

Note.

*p < .05

**p < .01

***p < .001

Mastery-avoidance goals are positive predictors of the extent of dynamic demands on plain roads, and negatives predictors of accelerations above 3 m/s^2^ on mountain roads (see Table 5). This means that a person who wants to avoid driving badly, uses the vehicle more dynamically on plain roads, and accelerates less on mountain roads.

**Table 5:** Results of the regression analysis for mastery-avoidance goals with the road context.

	Extent of dynamic demands in plain		Accelerations >300m/s^2^ mountainous	
	***R***	***R* ^2^**	***R***	***R* ^2^**
	.693	.480	.*239*	.*057*
	**Adj R^2^**	**Std error**	**Adj R^2^**	**Std error**
	.464	1.051	.*028*	.*157*
**Model: MAV**	***β***		***β***	
Gender	.029		-.109	
Age	.091		-.100	
Years of driving license	-.057		-.005	
Driving time	.683***		.022	
**Mastery-avoidance goals**	.112*		-.184*	

Note.

*p < .05

**p < .01

***p < .001

Finally, the results showed that performance-avoidance goals are positive predictors of the extent of dynamic demands in plain, and also in urban and extra-urban areas (see Table 6). In other terms, an individual who avoids being the worst driver uses the vehicle more dynamically on plain, in urban and extra-urban areas.

**Table 6:** Results of the regression analysis for performance-avoidance goals with the road context.

	Extent of dynamic demands in plain		Extent of dynamic demands extra-urban		Extent of dynamic demands urban	
	***R***	***R*** ^2^	***R***	***R*** ^2^	***R***	***R*** ^2^
	.693	.480	.*714*	.*510*	.*637*	.*406*
	**Adj R^2^**	**Std error**	**Adj R^2^**	**Std error**	**Adj R^2^**	**Std error**
	.464	1.051	.*495*	.*937*	.*388*	*1*.*065*
**Model: PAV**	***β***		***β***		***β***	
Gender	.029		.044		.061	
Age	.091		.012		-.064	
Years of driving license	-.057		.073		.028	
Driving time	.683***		.706***		.629***	
**Performance-avoidance goals**	.154**		.027*		.141*	

Note.

*p < .05

**p < .01

***p < .001

## Discussion

This study is the first to relate achievement goals to objective measures of driving behavior. It improves and expands the understanding of drivers’ psychological characteristics and driving behavior. This study showed the predictive role of achievement goals on some objective driving behaviors: incidents, percentage of time spent over an acceleration threshold, and extent of dynamic demands. In general, mastery-approach goals were found to be negative predictors of the extent of dynamic demands. By focusing on the road context, this relationship is also observed in plain. These goals are also negative predictors of heavy braking (i.e., < -3 m/s^2^), particularly on plain and hilly roads. Feeling in control of the driving task does not result in hard braking on plain and hilly roads nor in erratic dynamic demands of one’s vehicle on plain roads. All these results confirm our hypotheses, and relations observed in other domains [25] in which mastery-approach goals are linked to positive consequences. From the results obtained in the present study, it appears that mastery-approach goals are mainly predictors of positive objective driving outcomes.

Our study also showed that performance-approach goals are significant positive predictors of high longitudinal acceleration (i.e., > 3 m/s^2^). This relationship is confirmed on hilly and plain roads. They are also positive predictors of heavy transversal accelerations on left turns on plain roads. For these individuals, making strong accelerations on hilly and plain roads and taking left turns quickly on plain roads is a way of showing their superiority to others. This is consistent with our hypothesis and with findings in other domains (i.e., sport, education, and work), in which performance-approach goals positively predict cheating and aggressive behaviors [55, 56]. This result supports those found in driving with self-reported measures [11], in which performance-approach goals positively predict aggressive violations, ordinary violations, and sensation seeking.

Furthermore, performance-approach goals are found to be negative predictors of the extent of dynamic demands on plain roads. For these individuals, using the vehicle more dynamically on plain roads is not a way to demonstrate their superiority to others. Hilly and mountainous roads are possibly more appropriate to show others their superiority in driving This relationship is surprising: these goals are linked to a positive consequence (i.e. low extent of dynamic demands on the plains), whereas in the driving literature [11] and in other domains, they are mostly linked to negative consequences [55, 56]. However, some studies have reported that performance-approach goals can be associated with positive consequences such as concentration or information processing [57–59]. This result is even more surprising, given that these goals are also linked to various negative consequences in this study, especially in plain. On the one hand, we measure the time spent in heavy accelerations (longitudinal and transversal), which is more a frequency value. On the other hand, we measure the extent of the accelerations (longitudinal and transversal) reached at least once, and reaching a heavy acceleration only once is sufficient to give a significant extent value. These two variables are different, so opposite relationships such as those observed in our study are therefore conceivable. The adoption of performance-approach goals negatively predicts various negatives outcomes in driving, and only one positive outcome, so we conclude that these goals are more related to negative behaviors in driving.

Our study also showed that performance-avoidance goals are positive predictors of the extent of dynamic demands in general, and more specifically on plain, urban and extra-urban roads. Higher dynamic demands can generate more risky driving situations. In the literature on achievement goals in work and education, these goals are often linked to negative outcomes such as anxiety, negative affect, and low performance [60, 61]. Similarly, these goals in driving predict self-reported aggressive violations [11]. These relations observed in our study are consistent with our hypotheses and those observed in others domains. This result could be explained by the fear of failure that is strongly related to performance-avoidance goals [62]. Fear of failure can induce sudden accelerations and braking, which can produce greater dynamics demands, particularly in road contexts in which comparison with others drivers might be important.

Mastery-avoidance goals were found to be positive predictors of the extent of dynamic demands on plain roads, and negative predictors of important accelerations on mountains roads. These goals are linked to a negative consequence (important dynamic demands on plain roads) and a positive consequence (low acceleration in the mountains). These results are consistent with our hypotheses and with the protective role observed in driving with self-reported measured [10, 11]. Moreover, they are in accordance with the observations of Baranik et al. [22] and Senko and Freund [63], where mastery-avoidance goals were linked to both positive consequences (e.g., interest, need for achievement, perceived competence) and negative consequences (e.g., competitiveness, anxiety, procrastination). These relationships can be explained, similar to performance-avoidance goals, by the fear of failure, which is related to mastery-avoidance goals [20, 64]. Fear of failure could induce erratic acceleration and braking on plain roads, which would cause heavy dynamic demands. In driving, failure can be symbolized by an accident. The mountainous context being rugged, a small driving error could lead to a dangerous situation. To reduce this risk, the driver would accelerate less in the mountains.

In addition, no significant relationship was found between incidents and achievement goals. This result can be explained by the fact that an incident is not only related to the driver’s behavior, but also to the other road users’ behaviors, and by the state of road infrastructure, which were not assessed in this study.

The study has several limitations that open up perspectives for future studies. First, the study was conducted in a single country (France), yet Hulleman et al. [65] showed that the achievement goals of individuals in collectivist countries (i.e., Eastern countries) are not linked to the same consequences as those in countries with a more individualistic culture (i.e., Western countries). Moreover, Özkan et al. [66] have shown that driving style differs from one country to another. A cross-cultural study would determine whether mastery-approach goals are predictive of positive outcomes in driving regardless of country. Secondly, our study was conducted with a sample of 266 drivers; it would be relevant to increase the sample size to be as representative as possible. In the SHRP-2 study [67], 3400 drivers were recruited. A larger sample would also provide sufficient data in regard to road types, relief, and events, even if our study was conducted on more than 53 732 hours of driving and 4 242 482 km. Thirdly, the acquisition frequency used to collect the data is 5Hz, which might be too low to detect every incident. Naude et al. [9] based their study on an acquisition with a frequency of 100Hz, which allows for better accuracy in data collection, in particular for detecting driving incidents, and the time spent over an acceleration threshold. Fourthly, the study was only conducted with car drivers, but motorized two-wheelers are also very involved in road accidents. They constitute 22.8% of road deaths in France [68] and 43% in South East Asia [69]. It would be interesting to conduct the same kind of analysis on two-wheelers, to highlight whether the relationships observed with motorists follow the same pattern. Finally, it is possible to induce achievement goals to investigate the effects on individuals’ behavior [70, 71]. We have observed that the mastery-approach goals are the most protective and predict positive objective behaviors in driving. Therefore, it would be relevant to induce mastery-approach goals in an attempt to change driving behavior of at-risk drivers, and ultimately to improve road safety. The intervention could take place during drivers’ learning phase and in some countries during the recovery of license points, in an attempt to change the behaviors of at-risk drivers by directing them towards mastery-approach goals. This could also be done during road safety campaigns, whether on television or on the road via street signs, by delivering a message that favours the adoption of mastery-approach goals. Promotion messages can be directed toward the possibility of success, toward personal challenges, to encourage effort and perseverance [70].

### Conclusion

Achievement goals theory has been widely studied since the 1980s [16, 72, 73] in various domains (education, sport, work, etc.), while only very recently have they been studied in the driving domain [10, 11]. Indeed, the driving domain contains all the characteristics of an achievement context [15]. In these two studies, achievement goals in driving have been related only to self-reported variables (i.e., violations, accidents, at-fault accidents, emergency maneuvers, sensation seeking). However, NDS studies measure objective driving behaviors under ordinary conditions, using in-vehicle recording devices. The aim of the present study was to assess the predictive role of achievement goals on objective driving behaviors. The results highlighted that mastery-approach goals were negative predictors of heavy braking on plain and hilly roads, and of the extent of dynamic demands on plain roads. They are also negative predictors of heavy transversal accelerations in right turn on hilly roads. Performance-approach goals are positive predictors of high acceleration on plain and hilly roads, and negative predictors of the extent of dynamic demands on plain roads. Performance-avoidance goals were positive predictors of the extent of dynamic demands on plain, extra-urban and urban roads. Therefore, mastery-approach goals appear to be the most protective goals. It may be relevant to promote the adoption of mastery-approach goals, so that drivers can adopt at safer driving style.

## Supporting information

S1 File (XLSX)
